# Heparan Sulfate Structure Affects Autophagy, Lifespan, Responses to Oxidative Stress, and Cell Degeneration in *Drosophila parkin* Mutants

**DOI:** 10.1534/g3.119.400730

**Published:** 2019-10-31

**Authors:** Claire Reynolds-Peterson, Jie Xu, Na Zhao, Casey Cruse, Brandon Yonel, Claire Trasorras, Hidenao Toyoda, Akiko Kinoshita-Toyoda, Jennifer Dobson, Nicholas Schultheis, Mei Jiang, Scott Selleck

**Affiliations:** * Department of Biochemistry & Molecular Biology, The Pennsylvania State University, University Park, PA, 16802 and; † Faculty of Pharmaceutical Sciences, Ritsumeikan University, 1-1-1 Nojihigashi, Kusatsu, Shiga, 525-8577, JAPAN

**Keywords:** heparan sulfate modified proteins, autophagy, mitophagy, *parkin*, presenilin

## Abstract

Autophagy is a catabolic process that provides cells with energy and molecular building blocks during nutritional stress. Autophagy also removes misfolded proteins and damaged organelles, a critical mechanism for cellular repair. Earlier work demonstrated that heparan sulfate proteoglycans, an abundant class of carbohydrate-modified proteins found on cell surfaces and in the extracellular matrix, suppress basal levels of autophagy in several cell types during development in *Drosophila melanogaster*. In studies reported here, we examined the capacity of heparan sulfate synthesis to influence events affected by autophagy, including lifespan, resistance to reactive oxygen species (ROS) stress, and accumulation of ubiquitin-modified proteins in the brain. Compromising heparan sulfate synthesis increased autophagy-dependent processes, evident by extended lifespan, increased resistance to ROS, and reduced accumulation of ubiquitin-modified proteins in the brains of ROS exposed adults. The capacity of altering heparan sulfate biosynthesis to protect cells from injury was also evaluated in two different models of neurodegeneration, overexpression of Presenilin and *parkin* mutants. Presenilin overexpression in the retina produces cell loss, and compromising heparan sulfate biosynthesis rescued retinal patterning and size abnormalities in these animals. *parkin* is the fly homolog of human *PARK2*, one of the genes responsible for juvenile onset Parkinson’s Disease. Parkin is involved in mitochondrial surveillance and compromising *parkin* function results in degeneration of both flight muscle and dopaminergic neurons in *Drosophila*. Altering heparan sulfate biosynthesis suppressed flight muscle degeneration and mitochondrial dysmorphology, indicating that activation of autophagy-mediated removal of mitochondria (mitophagy) is potentiated in these animals. These findings provide *in vivo* evidence that altering the levels of heparan sulfate synthesis activates autophagy and can provide protection from a variety of cellular stressors.

Heparan sulfate modified proteins are abundant proteins of the cell surface and extracellular matrix, named for the unbranched and highly sulfated disaccharide polymers covalently attached to the protein core (Bishop *et al.* 2007; Esko and Selleck 2002; Lindahl *et al.* 2017). Biosynthesis of heparan sulfate occurs in the golgi and mutations affecting this enzyme machinery compromise the modification of numerous proteins, including glypicans and syndecans, two integral membrane proteoglycans involved in signaling. Studies of genes encoding proteins required for heparan sulfate polymer synthesis and sulfation have been instrumental in defining the activities of heparan sulfate modified proteins. These genes are highly conserved across species, including *C. elegans*, *Drosophila*, the mouse, and humans. Heparan sulfate modified proteins are functionally diverse molecules, regulating growth factor signaling, endocytosis, and the distribution of molecules in the matrix. Both the protein core and the heparan sulfate chains govern these functions. The abundance and broad expression of heparan sulfate modified proteins, together with their diverse functions in modulating signaling, provide the capacity to effect cellular physiology in a myriad of ways. Here we explore their function in regulating autophagy.

Autophagy is important for proteostasis, organelle turnover, and protecting cells from a variety of cellular stresses. In vertebrates basal autophagy is critical for normal cellular health, highlighted by the extensive neuronal death in the cerebrum and cerebellum of mice lacking Atg7, a critical autophagy component (Komatsu *et al.* 2006b). Upregulation of constitutive autophagy increases lifespan in *C. elegans (*Kapahi *et al.* 2017*)* and *Drosophila*(Simonsen *et al.* 2008) and can rescue neurons from protein-aggregate toxicity in a number of models, including *Drosophila* (Wang *et al.* 2009; Kim *et al.* 2017). Recent work has demonstrated that increases in basal autophagy regulated by Beclin can also increase lifespan and health span in the mouse (Fernández *et al.* 2018). There is also evidence that mitophagy, a component of autophagy, is important for removing damaged mitochondria and failure of mitochondrial surveillance has a significant role in the pathology of Parkinson’s disease (de Vries and Przedborski 2013).

In the course of examining heparan sulfate modified protein function at the *Drosophila* neuromuscular junction we discovered that heparan sulfate synthesis in muscle had profound effects on autophagy. Decreasing heparan sulfate synthesis produced reductions in the number of mitochondria and changes in the structure of post-synaptic specializations, effects shown to be mediated by an increase in autophagy (Reynolds-Peterson *et al.* 2017). The capacity of heparan sulfate modified proteins to suppress autophagy was also documented in fat body, a critical metabolic sensing and energy storage tissue in *Drosophila*. There are a number of findings in the mouse that suggest heparan sulfate modified protein-mediated inhibition of autophagy occurs in vertebrates as well. When heparan sulfate accumulates, such as in many lysosomal storage diseases, autophagy is suppressed (Fiorenza *et al.* 2018; Cox and Cachon-Gonzalez 2012; Bartolomeo *et al.* 2017; Settembre *et al.* 2008a). Transgene-mediated expression of a heparan sulfate-degrading enzymes, heparanase (Hpa1), increases autophagy, consistent with an inhibitory role of heparan sulfate modified proteins on autophagy (Ilan *et al.* 2015; Shteingauz *et al.* 2015). Conversely, gene knockout of *Hpa1* results in suppression of autophagy in multiple tissues, consistent with an inhibitory role of heparan sulfate modified proteins on autophagy levels.

However, specific heparan sulfate-modified proteins have been shown to affect autophagy in distinct manners. Loss of Perlecan increases autophagy in mouse muscle, consistent with an autophagy-inhibitory activity (Ning *et al.* 2015). However, Endorepellin, a C-terminal fragment of Perlecan, and Decorin, a small leucine-rich proteoglycan, induce autophagy (Gubbiotti and Iozzo 2015; Poluzzi *et al.* 2014). These studies demonstrate that individual proteoglycans can either inhibit or stimulate autophagy in differenct cellular contexts. We were interested in the effects of heparan sulfate modified proteins generally on autophagy regulation, and known physiological functions of autophagy, including responses to stress, lifespan, proteostasis, and mitophagy in an intact animal system.

In the studies reported here we examined the influence of heparan sulfate biosynthesis levels and sulfation state on known physiological functions of autophagy. We show that decreasing heparan sulfate levels or sulfation has all the hallmarks of global activation of basal autophagy, increasing resistance to oxidative stress and extending lifespan. We have also examined the capacity of altered heparan sulfate biosynthesis to provide protection from cell loss in a Presenilin model of Alzheimer’s Disease (AD), or deficits in mitochondrial surveillance mediated by mutations in *parkin*, the homolog of *PARK2*. In both of these models of human neurodegenerative disorders, altering heparan sulfate biosynthesis rescued cell loss, showing that changes in heparan sulfate can affect the capacity of cells to tolerate a variety of cellular stresses.

## Methods and Materials

### Fly rearing and strains

Fly strains were raised on standard cornmeal/sucrose/agar media at 25°. Oregon-R, *UAS-w*-RNAi (30033), and VDRC60100 strains served as controls; stock numbers are listed in parentheses. Unless otherwise specified, when *UAS*-*sfl*-RNAi is shown, *UAS*-*sfl*-RNAi HMS00543 (34601) strain was used. RNAi strains and a control strain with the same genetic background were obtained from the Vienna *Drosophila* RNAi Center (VDRC): *UAS-Atg8a*-RNAi (43097), *UAS-sfl*-RNAi (5070), *UAS-ttv*-RNAi (4871), *UAS-w*-RNAi (30033) and empty vector control (60100). *UAS-Atg5*-RNAi is Bloomington Stock number 27551.

*UAS-mcherry-Atg8a* (37750), *elav*^*C155*^*-Gal4* > *UAS*-*DcrII* (25750), *UAS-Atg8a* (10107), UAS-*Psn^541^* (8309) and the Drosophila Transgenic RNAi Project (TRiP) lines UAS-*sfl*-RNAi GLC01656 (50538), UAS-*sfl*-RNAi HMS00543 (34601), and UAS-*mCherry*-RNAi (35785) were obtained from the Bloomington Drosophila Stock Center (BDSC). The *sfl*^*03844*^ and *ttv*^*00681*^ *P*-element insertion alleles were generated by the Berkeley *Drosophila* gene disruption project and have been previously described. The *sfl*^*9B4*^ ethylmethanesulfonate-induced point mutation was kindly provided by Norbert Perrimon (Lin and Perrimon 1999). *parkin* alleles (*park^1^*, *park*^*Δ21*^) were obtained from the Bloomington Stock Center. *sfl** park^Δ21^* recombinant chromosomes were generated and the presence of *sfl* and *parkin* alleles determined by back crossing to lethal *sfl* and *parkin* alleles, as well as by PCR analysis of recombinant animals and QPCR to measure mRNA levels (see Supplemental Figure 2). Four recombinants of the two different *sfl* alleles were generated and evaluated. *botv*^*423*^ is a point mutation and loss-of-function allele (Takei *et al.* 2004). *UAS-mitoGFP* (*P*-element insertion on the second chromosome, Bloomington stock number 8442) was used to selectively tag mitochondria and muscle specific expression was achieved using *mef2-Gal4* (y[1] w[*]; P{w[+mC]=GAL4-Mef2.R}3 on the third chromosome, Bloomington stock number 27390).

### Lifespan and log-rank analysis

A typical experiment began with over 100 adult flies, 25-30 per vial, examined for viability on standard media at 25°, and transferred to new media every 48 hr. Significant deviation in survival curves was calculated first on the pooled genotypes by logrank test using the Mantel-Haenszel method, followed by pair-wise comparison between each experimental genotype and the control using the Gehan-Breslow-Wilcoxon test. No individuals were censored during the survival assay, and survival curves of the experimental genotypes do not cross that of the control.

### Oxidant exposure

Hydrogen peroxide toxicity was performed to observe ROS sensitivity. One-week old flies were placed in vials at 25°, 20 per vial, and with food media made of 1% sucrose, 1% dry yeast extract, 1.2% agar (w/v), and 1.5% hydrogen peroxide (Cumming *et al.* 2008). In addition to the ROS exposed set, a control set using the same conditions in the absence of hydrogen peroxide was done for each genotype. Mortality was scored every 12 hr and the media was replaced every 24 hr.

### Flight assay

Flight assays were conducted according to previously published protocols (Kawasaki *et al.* 2016). Briefly, adult flies are introduced into the top of a large diameter cylinder coated with oil. Flies stick to the wall of the cylinder when they land and the height of their landing provides a measure of their capacity to maintain flight; flightless animals fall to a pool of oil at the bottom of the cylinder.

### Immunohistochemistry and confocal analysis

Whole-mount immunostaining of adult flight muscles was carried out according to described procedures to visualize both ubiquitin levels and the organization of actin filaments (Hunt and Demontis 2013). Images were acquired at room temperature using an Olympus Fluoview FV1000 laser-scanning confocal microscope (Olympus America, Waltham, Massachusetts, USA). FV10-ASW 2.1 software (Olympus, Waltham, Massachusetts, USA) was used to capture images. When more than one fluorophore was detected, sequential line scanning was performed to avoid spectral bleed through artifacts. Images of samples with different genotypes within a single experiment were captured, processed, and analyzed using the same settings. Images were presented as Z-stacks of maximum intensity projections using Imaris 7.3 software (Bitplane Inc.). All adjustments to contrast and brightness made to ease interpretation of confocal images were applied identically to all genotypes within each experiment.

### Image analysis of ubiquitin-positive intracellular punctae in flight muscle

ImageJ was used to identify and measure the number of anti-ubiquitin antibody-positive punctae in the adult flight muscles of *sfl park ^Δ21^/mef2-Gal4 park^1^* and *UAS-Atg5^RNAi^/+*; *sfl** park ^Δ21^/mef2-Gal4 park^1^* animals. Animals were reared at 25° for the experiments illustrated in Figure 9. The presence of the *UAS-Atg5^RNAi^* adversely affected survival of *sfl** park ^Δ21^/mef2-Gal4 park^1^* (less than 10% of expected, n = 733). For every image, the brightness of the color threshold was set to a minimum of 70, to identify the punctae over background. The “Measure” function was used to determine the area and the “Particles” function was used to count the number of particles, with the circularity of the particles set to the restraints of 0.80-1.00. The “Exclude on Edges” function was enabled to remove extraneous staining at the edges of the preparation, and the number of punctae per unit area was determined. Differences between punctae/area for the genotypes was assessed using a two-tailed *t-test*. A replicate experiment conducted at a lower temperature (23°), where *Gal4* activity is less, and hence RNAi of *Atg5* lower, allowed for improved survival of *sfl** park ^Δ21^/mef2-Gal4 park^1^* animals. The presence of *UAS-Atg5^RNAi^* increased the number of ubiquitin-positive punctae in *sfl** park ^Δ21^/mef2-Gal4 park^1^* animals reared at this temperature but reducing the brightness thresholds to 40 was required to detect the intracellular punctae. The rectangle tool was used to select a 60-pixel by 60-pixel sampling area for each preparation and the circularity set to 0.7 – 1.0. *UAS-Atg5^RNAi^/+*; *mef2-Gal4 park^1^/+* animals served as controls to determine the interaction between *Atg5*^*RNAi*^ and *park* function. Elevated levels of punctae required two mutant alleles of *park* (*sfl** park ^Δ21^/mef2-Gal4 park^1^*).

### Eye morphometry and presenilin overexpression experiments

*UAS*-*Psn* is a 541 amino acid wildtype *Presenilin* coding sequence. This construct was overexpressed pan-neuronally under the control of *elav^C155^-Gal4* > UAS-*DcrII*. This Gal4-expressing line also incorporates UAS-based overexpression of *DcrII*, which is needed to elicit effective RNAi in neurons. The UAS-*Psn* construct was expressed on its own and in combination with several RNAi lines. Eyes were imaged using an Olympus BX53 compound microscope and Olympus cellSens Dimension software (Olympus Optical, Waltham, MA) to obtain optical sections at a 9μm step. Optical sections for each animal were assembled into a single composite image using Zerene Stacker (Zerene Systems LLC, Richland, WA). Eye area was measured in ImageJ and ommatidial organization was assessed using the Flynotyper ImageJ plugin (http://flynotyper.sourceforge.net/)(Iyer *et al.* 2016). Statistical analysis of the results were performed using GraphPad Prism software (version 8.1.2). First, D’Agostino and Pearson normality testing was performed for all genotypes, separated by gender. Kruskal Wallis non-parametric testing was then used since all samples were not normally distributed to determine if there were any significant differences between the groups. This pooled testing was followed by Uncorrected Dunn’s Test for pairwise comparisons, providing the p-values provided for the scatter plots in Figure 5.

### mCherry conversion assay

Pan-neuronal expression was achieved using *elav*C155*-Gal4* with *UAS-DcrII* to enhance RNAi efficacy and the *UAS-mCherry-Atg8a* reporter construct. *UAS-w-*RNAi was used as a control. Overexpression of wildtype Atg8a was used as a positive control for enhanced autophagy. Density of the parental mCherry-Atg8a band was divided by density of the free mCherry band to determine the ratio of fluorophore conversion. For the mCherry conversion assay, 30-60 adult heads were homogenized by ceramic bead agitation using the using a Bead Ruptor 24 (Omni International, Kennesaw, Georgia, 73 USA) in 100μl of SDS extraction buffer (2% SDS, 50mM Tris pH 7.4, 1X protease inhibitor cocktail Complete [Roche, 10184600]). Samples were spun down at 10,000rpm for 10 min and the supernatant was removed to a clean tube.

### Insoluble ubiquitin protein assay

A two-step protein extraction was used for Insoluble Ubiquitin Protein analysis, modified from a previously described method (Cumming *et al.* 2008). Typically, 30-60 adult heads were homogenized by ceramic bead agitation using the Bead Ruptor 24 in 150ul of Triton-X100 extraction buffer (1% Triton-X100, 1x PBS, 1X protease inhibitor cocktail Complete [Roche, 10184600]). Samples were spun down at 10,000 RPM for 10 min at 4°. The supernatant was drawn off and saved as the soluble protein fraction. The pellets were washed with 100ul of TritonX-100 before being resuspended in 100ul of 2% SDS extraction buffer. Supernatant from this extraction was saved and is referred to as the TritonX insoluble fraction. Protein sample concentration was determined by BCA Protein Assay (Pierce, 23227).

### Western blotting

IUP assessment utilized 9% acrylamide gels, while the analysis of mCherry-Atg8a utilized 6% acrylamide gels. PAGE was performed using the BioRad mini-PROTEAN electrophoretic and transfer system (BioRad, Hercules CA) with 1mm plates. Protein samples were prepared for loading and membranes were processed as described above. Primary antibody incubation was performed overnight at 4° using 1:2000 mouse anti-Mono- and Polyubiquitinylated Conjugates (FK2) (Enzo, BML-PW8810-0100) or 1:2000 rabbit anti-mCherry (abcam, ab183628), and 1:3000 mouse anti-tubulin (Developmental Studies Hybridoma Bank, 12G10). Secondary antibody incubation (1:3000 HRP conjugated goat anti-mouse or –rabbit, Invitrogen, 31430 and 7431460) was performed for 45 min at room temperature. ECL detection was performed using G:BOX Chemi XG4 (Syngene, Fredrick, Maryland, USA) which was also used to invert coloration of resulting images. Densitometry was performed using ImageJ 1.42q. For loading analysis, a combination of Ponceau staining and stripping and reprobing with anti-alpha tubulin was used. Membranes were stripped for re-probing in mild stripping buffer (0.1% SDS, 1.0% Tween20, 0.2M glycine, pH 2.2). All blotting experiments were performed using three biological replicates for each genotype under each condition. Lanes with the most numerically similar sample loading, as determined by densitometry, were chosen from within the biological triplicates for display.

### Quantitative reverse transcription PCR

QPCR assays of *sfl*, *ttv* and *parkin* were carried out to evaluate the levels of these transcripts in animals heterozygous for *sfl* or *ttv* alleles and either heterozygous or homozygous for *park* alleles. Transcript levels were also measured in larvae bearing *UAS-sfl^RNAi^* or *UAS-ttv^RNAi^* expressed under the control *da-Gal4*.

RNA was isolated from 20 flies per sample with a Machery-Nagel DNA, RNA and Protein Purification Kit. RNA was diluted to 200 ng/ul then used in a High Capacity cDNA Reverse Transcription Kit (Applied Biosystems). This was put through an Eppendorf Mastercycler Pro PCR machine to create cDNA. Real time PCR was performed in a StepOne machine, using TaqMan assays for *Drosophila* gene targets *park*, *sfl*, *ttv*, and *arginine kinase* (Thermo Fisher Scientific).

Data were analyzed using Thermo Fisher Connect software to determine the relative quantifications. Three biological replicates with two technical replicates were used for each genotype. Target gene data were normalized to arginine kinase using the ΔΔCt method.

### Statistical analysis

Statistical analyses of quantitative data were performed using either Minitab Release 16 (Minitab) or GraphPad Prism 8.1.2 (GraphPad Software). Data were represented as the mean ±95% confidence interval unless otherwise indicated. Data distributions were tested using D’Agostino &amp;amp; Pearson normality test. Comparisons between more than two groups were performed using ANOVA or Kruskal-Wallis for nonparametric and or heteroscedastic data, followed by individual pairwise comparisons using Dunn’s test for non-parametric data or a *t*-test for normally distributed data.

### Data availability

Strains and plasmids are available upon request. All other data necessary for confirming conclusions of the study are present with the article, figures and table. Supplemental material that includes, Table S1: Analysis of heparan sulfate levels and structure in *sfl* and *ttv* heterozygotes, Figure S1: Frequency distribution of pixel intensities for anti-ubiquitin staining in flight muscles of *parkin* mutants and *ttv**/+*; *park*^*Δ21*^*/park^1^*animals, Figure S2: QPCR analysis, Figure S3: Original western blots used for assembly of Figure 4, are available at figshare: https://doi.org/10.25387/g3.9989801.

## Results

### RNAi of genes required for heparan sulfate biosynthesis increase autophagy-dependent cleavage of Atg8a in the brain

**Figure 1:**
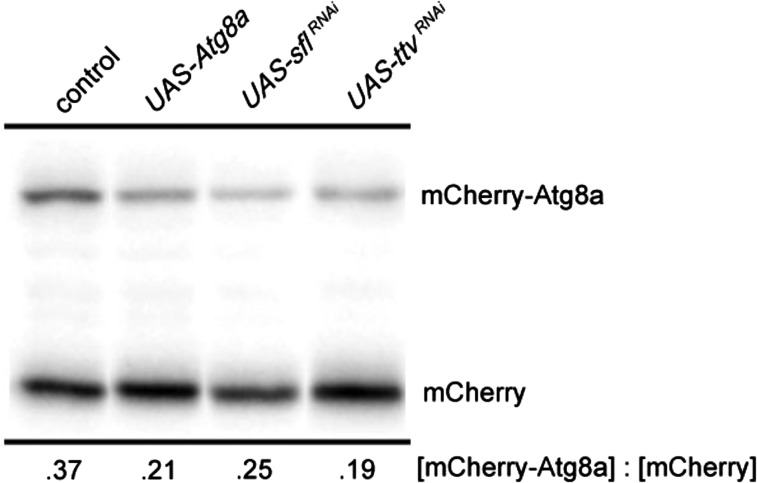
Autophagy-dependent cleavage of mCherry-Atg8a is increased in the CNS upon RNA interference of *sfl* or *ttv*. Heads from adult animals bearing transgene constructs expressing an mCherry-Atg8a fusion protein in neurons were obtained and mCherry-Atg8a protein detected by SDS-PAGE and western blotting with anti-mCherry antibody. The large fusion protein is cleaved during autophagosome maturation to a relatively stable product that contains the mCherry epitope. The ratio of the parental and cleavage products provides a measure of autophagy-dependent activity in the CNS. *UAS-w^RNAi^* was used as the control sample. Activation of autophagy in neurons with overexpression of Atg8a shows increased relative levels of the mCherry-bearing cleavage product. Knockdown of either *sfl* or *ttv* using RNAi also produces an increase in the relative levels of mCherry-Atg8a cleavage in the brain. Ratios provided below the image are averages of 4 samples, 2 replicates in two separate experiments. QPCR of *sfl* mRNA showed reductions to 42% of wild type levels (data not shown).

Earlier work demonstrated that reducing heparan sulfate biosynthesis increased autophagy in muscle and fat body, the latter being the principle metabolic sensing and energy storage tissue in *Drosophila* (Reynolds-Peterson *et al.* 2017). Given the critical role autophagy plays in neurodegenerative processes we wanted to determine directly if heparan sulfate biosynthesis affects autophagy in the brain. Autophagy-mediated proteolysis produces cleavage of key components, including Atg8a, a protein involved in autophagosome formation. Monitoring Atg8a cleavage is therefore one measure of autophagy. To evaluate Atg8a proteolytic processing we expressed an mCherry-Atg8a fusion protein in the brain using a UAS-mCherry-Atg8a construct and a neuron-specific Gal4 transcriptional activator. The resistance of the mCherry domain of the fusion protein to degradation allows the visualization of a relatively stable proteolytic product detected with an anti-mCherry antibody. The ratio of the parental protein, mCherry-Atg8a, to the mCherry proteolytic product provides a measure of autophagy-dependent activity (Figure 1)(Klionsky *et al.* 2016). As previously reported, activation of autophagy by transgene expression of Atg8a produced greater conversion of the mCherry-Atg8a fusion protein to the smaller mCherry-containing fragment. We examined the effect of heparan sulfate structure on Atg8a proteolytic cleavage by knockdown of either of two genes required for heparan sulfate biosynthesis, *sulfateless** (sfl)* or *tout velu* (ttv). *sfl*, encodes *N*-deacetylase *N*-sulfotransferase, an enzyme affecting sulfation of the heparan sulfate polymer (Toyoda *et al.* 2000a; Toyoda *et al.* 2000b). *ttv* encodes a homolog of Exostosin 1 (Ext1), a glycosyl transferase critical for heparan sulfate chain elongation. RNAi of either *sfl* or *ttv* promoted proteolytic cleavage of mCherry-Atg8a, indicative of a net increase in autophagy in the CNS (Figure 1).

### Reductions of heparan sulfate biosynthetic gene function increase lifespan

**Figure 2:**
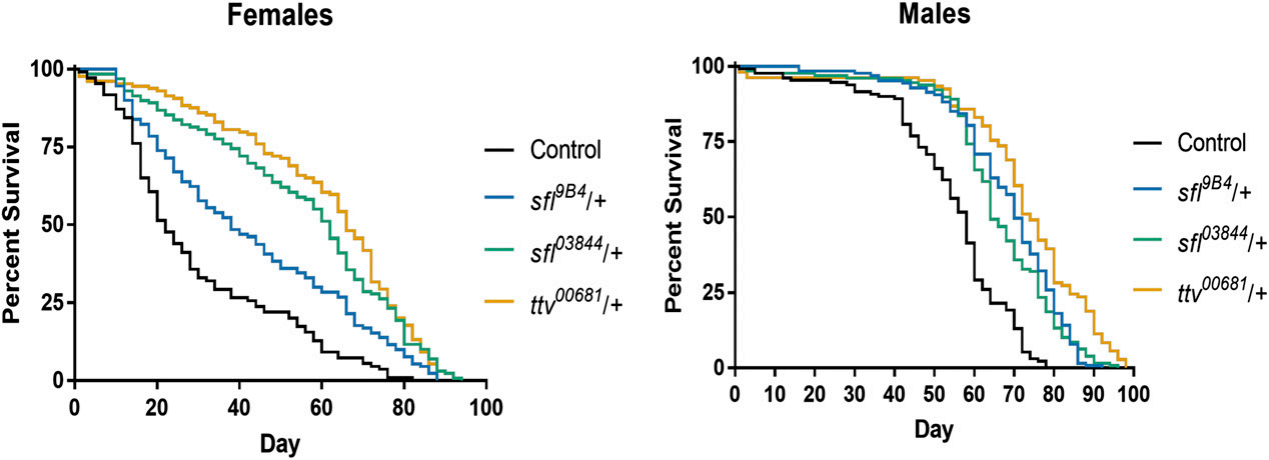
Reducing function of heparan sulfate biosynthetic genes increases lifespan. Animals heterozygous for alleles of either *sfl*, or *ttv* were examined for lifespan under normal culture conditions. Reductions in the functions of either of these heparan sulfate biosynthetic genes significantly extended lifespan (*P* < 0.0001 compared to control for each gender and genotype).

Increases in autophagy can extend lifespan, and longevity therefore provides a measure of autophagy function in the organism (Kapahi *et al.* 2017; Kinghorn *et al.* 2016; Wang *et al.* 2016). Lifespan was evaluated in adult animals heterozygous for mutations in *sfl* or *ttv*, each affecting different components of the heparan sulfate biosynthetic apparatus. Reducing the function of either of these two genes provided significant extension of lifespan compared to wild type control animals in both males and females (Figure 2). Overall *ttv**/+* appears to increase lifespan to a greater degree than *sfl**/+* animals. We do not know the basis of these differences but heterozygosity for mutations in these genes does affect the levels and structure of the heparan sulfate polymer differently (see below and Supplemental Table S1), perhaps accounting for the differential effects on lifespan. Together, these data provide evidence of the systemic impact of heparan sulfate modified proteins on the physiology of the organism consistent with an increase in autophagy levels.

Given the effect of heterozygosity for genes required for heparan sulfate biosynthesis on lifespan it was of interest to determine the structure and levels of the heparan sulfate polymer in adult animals bearing these genotypes. Disaccharide profiling of heparan sulfate derived from *ttv**/+* and *sfl**/+* animals compared to wild type controls was conducted using a method that provides both levels and composition of six disaccharides that comprise the polymer(Toyoda *et al.* 2000a; Toyoda *et al.* 1999). These analyses demonstrated that reducing the gene function by approximately 50%, with heterozygosity for alleles of either *sfl* or *ttv*, had a detectable and significant effect on both the quantity and sulfation state of the heparan sulfate polymer. In two different and independently isolated *sfl* alleles, heterozygosity lowered the levels of heparan sulfate-derived disaccharides by approximately 20% (Table S1) and also altered the sulfation pattern, reflected in the levels of different mono, bi and tri-sulfated disaccharides. Two disaccharides, the monosulfated NS(ΔUA-GlcNS), and the disulfated 2SNS (ΔUA2S-GlcNS), were affected the most, reduced by approximately 20% compared to wild type animals. Animals heterozygous for a *ttv* null allele, showed lower amounts of all disaccharides, to levels between 65–79% of wild type but without remarkable changes in the sulfation pattern. Previous analysis of third instar larvae heterozygous for *ttv* showed similar effects(Toyoda *et al.* 2000a). These results are consistent with the established activities encoded by *sfl* and *ttv*.

### Reducing heparan sulfate biosynthetic capacity increases resistance to ROS stress

**Figure 3:**
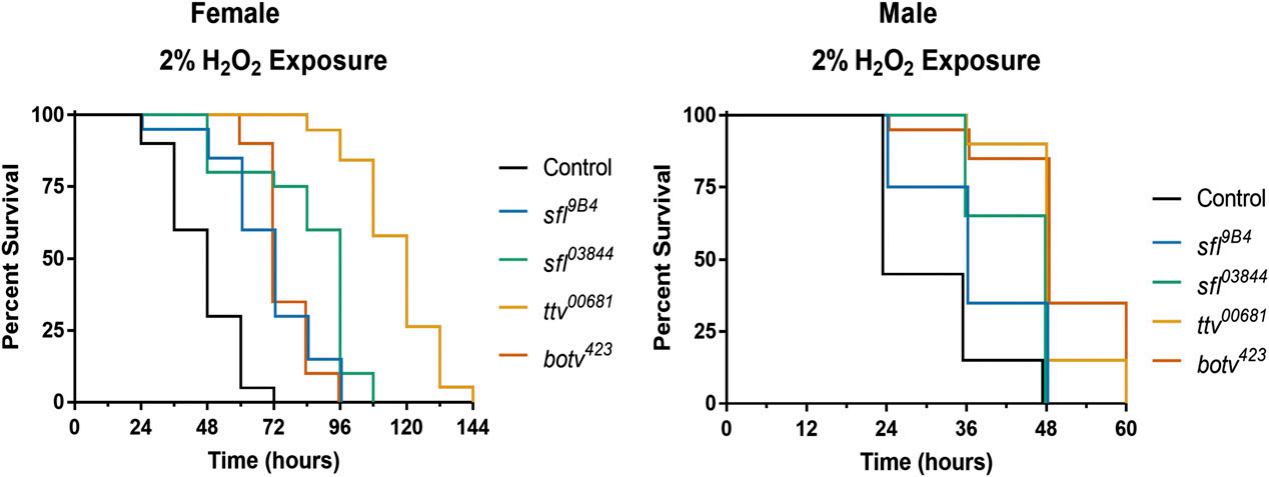
Reduced function of key heparan sulfate biosynthetic genes increases tolerance of oxidative stress. Adult flies were exposed to food containing 2% H_2_O_2_. Heterozygosity for mutations in any of three heparan sulfate biosynthetic genes improved survival to this challenge (Females, compared to control: *P* < 0.0001 all genotypes. Males, compared to control, wild type adults: *P* < 0.05 *sfl*^*9B4*^; *P* < 0.001 *sfl*^*03844*^, *ttv*^*00681*^, *botv*^*423*^). A typical experiment began with approximately 150 flies, 25-30 per vial.

Increasing autophagy provides protection against exposure to reactive oxygen species (ROS) stress by elevating the capacity of cells to remove damaged macromolecules. Given the broad increases in autophagy measured in whole larvae, larval muscle and fat body, as well as adult brain upon reduction of heparan sulfate biosynthesis we wanted to determine if organism-wide decreases in heparan sulfate gene function could affect responses to oxidative stress. Measures of resistance to ROS exposure were therefore conducted for animals heterozygous for mutant alleles of *sfl*, *ttv*, and *brother of ttv (botv)*. *botv* encodes the homolog to vertebrate *EXT Like-3*, an *N*-acetylglucosamine transferase-II required for heparan sulfate synthesis. To examine ROS sensitivity, adult flies were continuously exposed to H_2_O_2_ in food media and their survival monitored. Reductions in heparan sulfate biosynthetic gene function significantly increased survival to ROS exposure for all animals heterozygous for mutations in either of these three heparan sulfate biosynthetic enzyme-encoding genes (Figure 3). In female animals, wild type controls had a median survival duration of 48 hr, while *sfl*^9B4^/+ or *sfl*^03844^/+ animals survived for a median of 72 and 96 hr respectively. Heterozygosity for *ttv* further extended median survival to 120 hr, while *botv*/+ animals survived for a median of 72h. Males were more sensitive to peroxide exposure than females, but heterozygosity for all of these alleles still significantly increased their tolerance. These findings indicate that compromising heparan sulfate gene function can have a significant impact on a whole-organism response to ROS.

### Knockdown of heparan sulfate biosynthetic enzyme encoding genes in the brain reduced levels of insoluble ubiquitin-modified proteins upon ROS exposure

**Figure 4:**
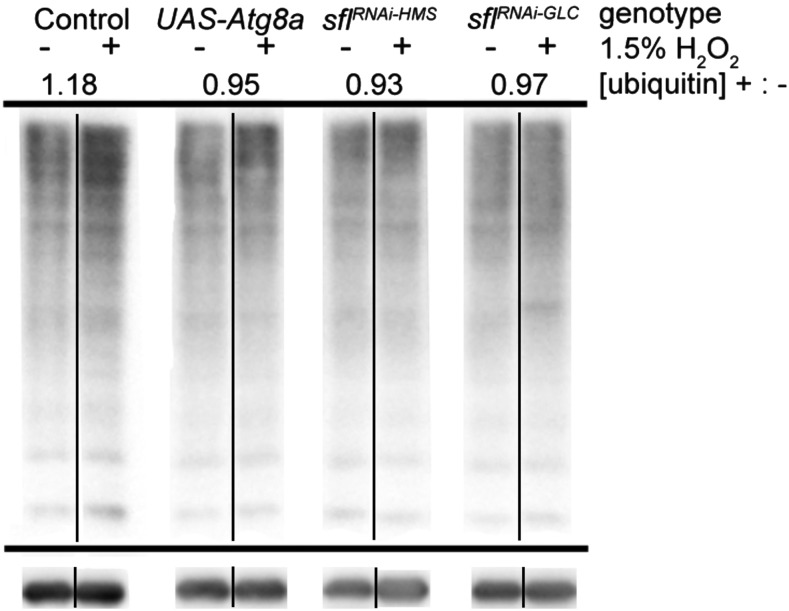
Reduction of heparan sulfate biosynthetic function prevents increases of ubiquitin-modified protein in the brains of ROS exposed animals. Accumulation of insoluble ubiquitinated proteins (IUPs) was examined in the triton-X100 insoluble fraction of total head proteins. *UAS-mCherry* RNAi, an shRNAi with no predicted targets in the *Drosophila* genome, was used as a control. All plus/minus oxidant pairs are from the same blot. All lanes are from the same experiment, with the same standard sample loaded on all gels from this one experiment for standardization (see Supplemental Figure 3 for original blots). Overexpression of Atg8a (*UAS-Atg8a*) was used as a positive control for enhancement of autophagy. The UAS-constructs were expressed under control of elav*-GAL4* with *UAS-dcrII* to enhance RNAi efficacy. Lanes from anti-ubiquitin stained membranes are shown in control *vs.* oxidant-exposed pairs. The ratio depicted is the average density of anti-ubiquitin staining, normalized to loading, in samples with peroxide (+) divided by samples without peroxide (-). Two different *sfl*^*RNAi*^ lines were tested. Two additional replicate experiments gave average peroxide/no peroxide ratios of 1.23 for control, and 0.8 for *sfl*^*RNAi-HMS*^. Two replicates of *ttv*^*RNAi*^ also showed lower levels of ubiquitin-insoluble material upon peroxide exposure (ratio of 0.7).

Ubiquitin-modified proteins are subject to targeted autophagic degradation through binding to the autophagy receptor p62. Particularly in mature adults, levels of autophagy in a tissue are generally inversely proportional to the amount of ubiquitin present. We assessed the levels of insoluble ubiquitin-modified proteins isolated from the brains of adult animals with CNS-directed RNAi of *sfl* or *ttv* after a 24-hour exposure to control or H_2_O_2_-containing food. Adult heads were isolated, the proteins solubilized, and the Triton X-100 insoluble fraction obtained according to published protocols(Simonsen *et al.* 2008). This fraction was separated by SDS-PAGE and ubiquitin-modified proteins detected by western blotting. This procedure has been used to detect age- and autophagy-dependent changes in the clearance of ubiquitin-modified substrates in the CNS of adult *Drosophila*. The levels of ubiquitin were measured for the entire lane for each sample and the levels normalized by comparison to the signal detected with anti-tubulin antibody. In control animals, ROS exposure increased the level of ubiquitin-modification of brain proteins, while increasing autophagy by overexpression of *Atg8a* reduced the levels of ubiquitin-modified proteins compared to control animals (Figure 4). In adult flies expressing either of two unique RNAi constructs to knock down *sfl* expression, the levels of insoluble ubiquitin-modified proteins were similarly reduced in oxidant-exposed animals compared to controls. These findings show that the level of key genes required for heparan sulfate biosynthesis have an impact on ROS-mediated accumulation of ubiquitin-modified proteins in the brain.

### Knockdown of sfl or ttv suppresses neurodegeneration mediated by overexpression of Presenilin

**Figure 5:**
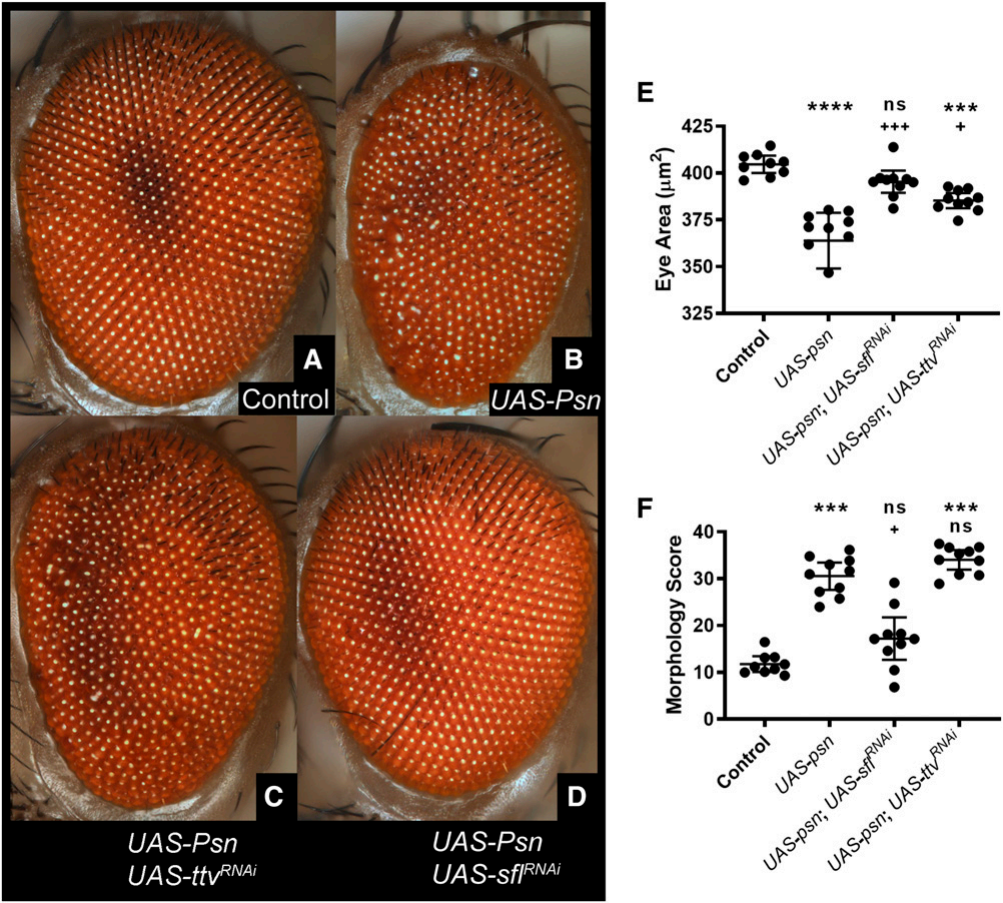
Reduction of *sfl* or *ttv* function rescues retinal abnormalities produced by overexpression of Presenilin. Transgene-mediated overexpression of Presenilin in neurons produces cell death and a reduction of the size of the eye, as well as disrupted patterning. Using brightfield illumination, serial optical sectioning and computational reconstruction, high-resolution images of the retina were obtained. Light reflection from each ommatidium (the photoceptive unit) provides the location and hence geometry of the retina. Expression of Presenilin (*elav-Gal4^c155^* > *UAS-Psn*) produces a rough and reduced retina (compare panels A and B; 9 animals for control, 10 animals for each experimental genotype), as well as disruption of the arrangement of ommatidia. The area of the retina provided a measure of cell loss (E). A computational method (Flynotyper) was used to obtain a measure of retinal disorganization, with higher scores representing increased disarray (F). RNAi of either *sfl* or *ttv* suppressed the effects of Presenilin overexpression on retinal size to a significant degree (compare panels B to C and D and measurements, panel E), whereas only *sfl* RNAi significantly rescued the disordering of the retina, measured by Flynotyper morphological scoring parameters (panel F). In panels E and F, data are presented as a scatter plot of each animal with the mean plus/minus 95% confidence interval. * (top row) indicate comparison to control, while + (bottom row) indicate comparison to Presenilin expression alone. */+ *P* < 0.05, **/++ *P* < 0.01, ***/+++ *P* < 0.001, **** *P* < 0.0001. Statistical testing utilized Kruskal-Wallis non-parametric group test followed by Dunn’s pairwise comparisons.

Reducing heparan sulfate biosynthetic function provided protection from ROS, extending survival to peroxide exposure. This enhanced survival was accompanied by reductions in ubiquitin-modified proteins in the brain, indicative of increased clearance of damaged proteins. We therefore wanted to determine if reductions of heparan sulfate biosynthesis were protective for neurotoxic stress. Missense mutations in Presenilins account for a sizable fraction of familial AD cases and models of Presenilin-mediated neurodegeneration have been established in *Drosophila*. Recent analysis of 138 pathogenic mutations in *PSEN1* demonstrate that approximately 90% compromise the function of the encoded γ-secretase (Sun *et al.* 2017) further supporting the hypothesis that neuronal loss is likely a consequence of this reduced function (Kelleher and Shen 2017). Overexpression of Presenilin (Psn) in *Drosophila* produces apoptotic and neurogenic phenotypes resembling Presenilin loss-of-function phenotypes, indicating this model provides some parallels with the human pathology (Ye and Fortini 1999). Presenilin overexpression in the *Drosophila* retina produces neuronal loss and patterning abnormalities. We have employed this model to determine if downregulation of heparan sulfate biosynthesis could affect neuronal loss and disruption of retinal patterning mediated by overexpression of Psn. A *Drosophila* Psn transgene was expressed under the direction of a neuron-specific Gal4 line, in the presence or absence of *UAS*- transgenes encoding double-stranded RNAi targeting either *ttv* or *sfl* mRNAs. Expression of *Psn* produces a marked reduction in the size of the adult eye as well as a disruption of patterning, seen in the disorganization of the facets of the retina (Figure 5, compare A and B). RNAi of *sfl* produced a significant rescue of both retinal size reduction and disordering (Figure 5, A,B,D, E and F). The degree of patterning defect was measured using Flynotyper (Iyer *et al.* 2016), an automated image processing algorithm that measures several features of retinal organization using the light reflected from each eye facet. Flynotyper calculates a single numerical score, reflecting several features of eye geometry. RNAi of *ttv* significantly rescued retinal size (Figure 5, panel C,E), represented as eye area, but did not improve the degree of retinal disorganization (Figure 5 F). Thus, compromising the function of two different genes in the heparan sulfate biosynthetic pathway reduced the developmental toxicity of Psn overexpression.

### Altering heparan sulfate structure rescues muscle degenerative phenotypes of parkin mutants

**Figure 6:**
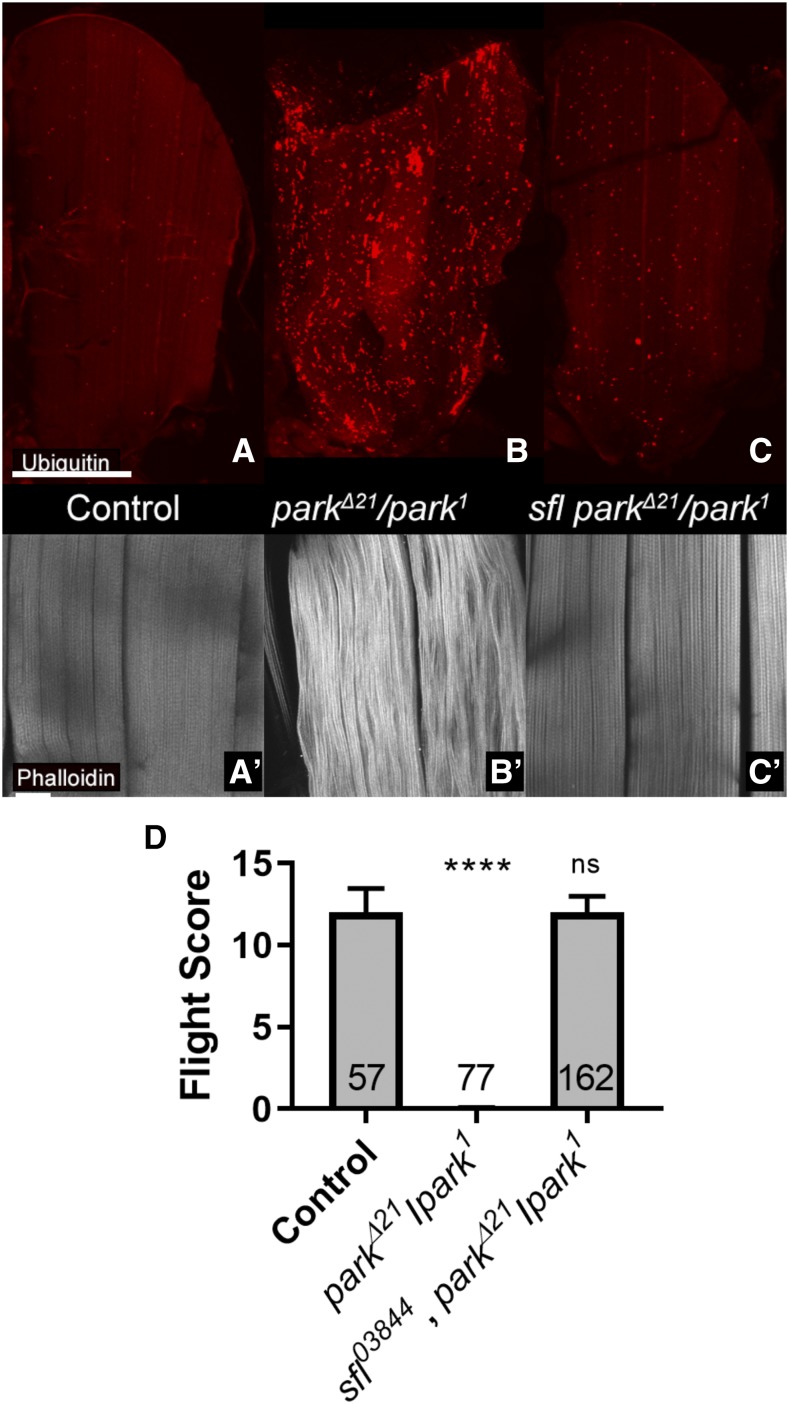
Reducing *sfl* function rescues muscle deficits found in *parkin* mutants. Animals homozygous for *parkin* alleles show flight muscle degeneration, accompanied by accumulation of ubiquitin and actin filament degeneration (compare panels A, A’ to B and B’). Panels A-C show low magnification confocal views of the indirect flight muscles of an adult fly, stained with anti-Ubi antibody. Panels A’-C’ show phalloidin staining of actin filaments from animals of the same genotypes as shown in panels A-C. Note the accumulation of ubiquitin in *park*^*Δ21*^*/park^1^* animals compared to controls and the marked decrease in ubiquitin in animals bearing these same *park* alleles as well as an allele of *sfl* (*sfl^03844^ park^Δ21^/park^1^*). Panel D shows flight assay results of control (*park^1^/+*, n = 57), park^*Δ21*^*/park^1^*mutants (N = 77), and *park*^*Δ21*^*/park^1^* mutants bearing a *sfl* allele (N = 162) (error bars showing standard error of the mean). **** *P* < 0.0001 Heterozygosity for *sfl* significantly rescued flight capability measured by this assay, from essentially no flight, to nearly wild type levels. Scale bars represent 200μM, 30 μM in A, and A’, respectively. Statistical testing utilized Kruskal-Wallis non-parametric group test followed by Dunn’s pairwise comparisons.

**Figure 7:**
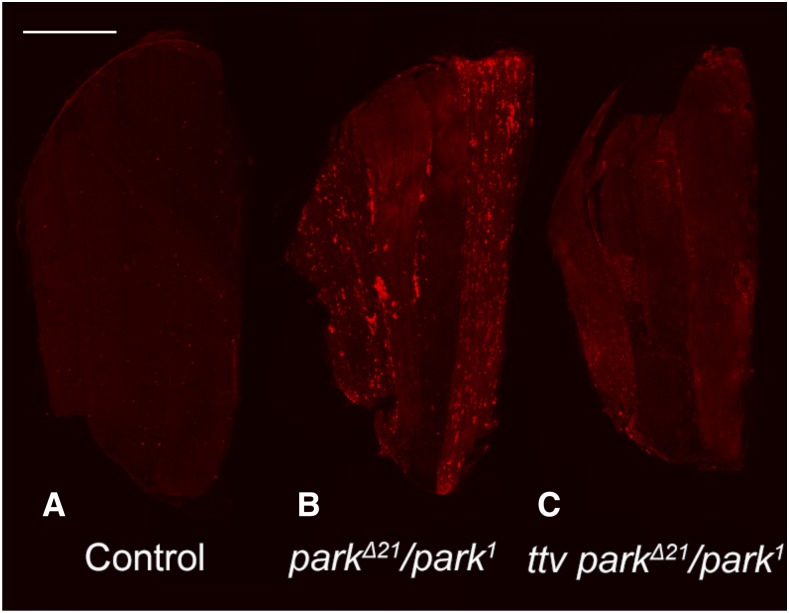
Reduction of *ttv* function rescues flight muscle abnormalities of *parkin* mutant animals. Adult flight muscles were dissected, fixed, stained with anti-ubiquitin antibody and visualized by confocal microscopy. *park*^*Δ21*^*/+* animals served as controls for this experiment. Note the reduced levels of anti-ubiquitin signal intensity in animals bearing a single allele of *ttv* (panels B *vs.* C). The frequency distribution of anti-ubiquitin signal (number of pixels for each pixel intensity level, see Supplemental Figure 2) was assessed for 15 animals of each genotype and analyzed using the Wilcoxon Signed Ranked Median Test. This assessment showed a significant difference (*P* < 0.005) between *park*^*Δ21*^*/park^1^* and *ttv*^*00681*^; *park*^*Δ21*^*/park^1^* animals. Scale bars represent 200μM.

Autophagy is responsible for engulfment and removal of damaged mitochondria, a function critical for mitochondrial quality control. Mutations in *parkin** (park)*, a *Drosophila* homolog of the human Parkin-encoding gene (*PARK2*), have been identified and animals bearing these mutations characterized (Burman *et al.* 2012; Pesah *et al.* 2004). Mutations in the human gene, *PARK2*, are responsible for a substantial fraction of familial Parkinson’s Disease patients, emphasizing the relevance of understanding PARK2/parkin function in the pathophysiology of this disorder (Giannoccaro *et al.* 2017; McWilliams and Muqit 2017). Studies of Drosophila *park* mutants have demonstrated that Park affects mitochondrial surveillance and autophagy-mediated removal (Pesah *et al.* 2004; Vincow *et al.* 2013). Loss of *park* produces degeneration of flight muscle, a highly metabolically active cell, and certain allelic combinations of *park* (*park*^1^/*park***^Δ^**^21^*)* survive to adulthood but are flightless, showing progressive muscle degeneration (Pesah *et al.* 2004). We examined the capacity of altering heparan sulfate biosynthesis to alter flight muscle cell degeneration in *park* mutants with the rationale that increasing autophagy could ameliorate the accumulation of damaged mitochondria. Heterozygosity for *sfl* had a profound effect and reduced the severity of *park* mutant phenotypes (Figure 6). Both *sfl*^*9B4*^ and *sfl*^*03844*^ alleles had similar effects on suppressing muscle cell abnormalities, including the accumulation of ubiquitin-modified proteins and disordering of actin filaments (Figure 6 A-C). Muscle function was evaluated with an assay that provides a quantitative assessment of flight. *park*^1^/*park***^Δ^***^21^* animals are incapable of flight (zero score) whereas *sfl* *park*^1^/*park***^Δ^***^21^* flies have near wild type levels (Figure 6D). Similar analyses were conducted for interactions between *ttv* and *park* and heterozygosity for a null allele of *ttv* also showed significant rescue of *park*^1^/*park***^Δ^***^21^* flight muscle abnormalities (Figure 7). Reduction of *ttv* function also reduced the levels of ubiquitin-modified proteins in the indirect flight muscle of *park* mutants (Figure 7 and Figure S1). Quantitative PCR was used to confirm that mutations in *sfl*, *ttv* and *park* (*park****^Δ^****^21^*) reduced the respective levels of mRNA (Figure S2) and predicted levels of mRNA were found in the animals that showed rescue of *park*-mediated phenotypes. The rescue of *park* mutant animals by reductions of *sfl* or *ttv* function provides evidence of the capacity of altered heparan sulfate synthesis to promote cellular repair systems that counteract cellular stresses, including accumulation of damaged mitochondria.

**Figure 8:**
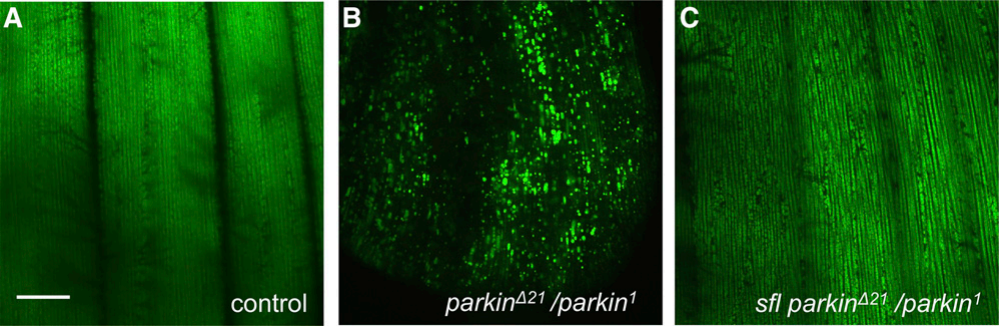
Reducing *sfl* function rescued mitochondrial abnormalities of *parkin* mutants. Mitochondria in adult flight muscle cells were tagged with *UAS-mitoGFP* expressed in muscle under the direction of a muscle-specific *Gal4* line, *mef2-Gal4*. This marker was crossed into *parkin* mutants and *parkin* mutants bearing a single mutant allele of *sfl*. Control animals (A) are wild type genotype, and B and C show *parkin* mutants without and with the *sfl* allele respectively. Representative images from two separate experiments are shown (n = 52). The scale bar represents 30μM.

Parkin functions in the surveillance and tagging of damaged mitochondria for removal by autophagic degradation. This is achieved via Parkin mediated ubiquitin-modification of outer membrane mitochondrial proteins, providing a molecular tag for recognition by the autophagy machinery. In accordance with the function of Parkin in mitochondrial surveillance, *parkin* mutants show accumulations of abnormal mitochondria. To determine if this critical and central phenotype of *parkin* mutants is affected by the levels *sfl* function we examined animals where mitochondrial were selective tagged by the expression of mito-GFP, a mitochondrial targeted protein, under the direction of a muscle-specific Gal4 line, mef2-Gal4. *parkin* mutants showed large and dysmorphic mitochondria compared to control animals and these changes were reversed by heterozygosity for *sfl* (Figure 8). These results establish that changes in heparan sulfate structure can modulate the cellular pathology of *parkin* mutants at the level of the primary deficit, failure to tag and removed damaged mitochondria.

#### Autophagy dependence of sfl-mediated rescue of cell degeneration in parkin mutants:

**Figure 9:**
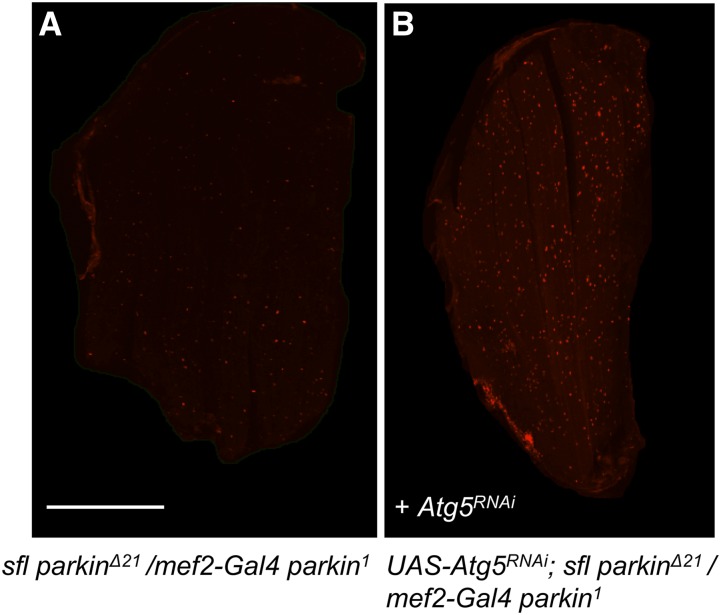
Autophagy dependence of *sfl*-mediated rescue of *parkin* flight muscle ubiquitin accumulation. RNAi of a critical autophagy gene, *Atg5*, resulted in increased intracellular ubiquitin, a hallmark of the cellular pathology observed in *park*^*Δ21*^*/park^1^* flight muscles; a phenotype which is rescued by *sfl* (see Figure 6). Panels A and B show confocal images of adult flight muscles stained with anti-ubiquitin antibody from *sfl** park^Δ21^/mef2-Gal4 park^1^* (A) or these same animals but bearing a *UAS-Atg5 ^RNAi^* transgene (B). *mef2-Gal4* provides muscle-specific expression of the *UAS-Atg5 ^RNAi^* transgene. The number of ubiquitin-positive punctae/pixel were measured and determined to be significantly different between these two groups using a two-tailed *t-test* statistic [*t=*6.49, *P* < 0.0001, n = 32]. Scale bars represent 200μM.

Alteration of heparan sulfate structure has a dramatic impact in two genetic models of cell degeneration in *Drosophila*, overexpression of Presenilin and *parkin* mutants. It is also evident that heparan sulfate structure can modulate autophagy in muscle, fat body, and neurons (Reynolds-Peterson *et al.* 2017) [data presented in this study]. To determine if autophagy function is required for the capacity of *sfl* to affect the rescue of cell degeneration in *parkin* mutants we used muscle-directed expression (*mef2-Gal4)* of *Atg5*^RNAi^. In *parkin* mutant animals ubiquitin accumulation is reduced by the presence of a single *sfl* mutant allele (Figure 6). When reared at 25°, a temperature where Gal4-directed transcription is active, RNAi of *Atg5* resulted in significantly increased accumulation of ubiquitin in the muscle cells of *sfl** park^Δ21^/mef2-Gal4 park^1^* adult animals (Figure 9). At this temperature, the knockdown of *Atg5* produced significant lethality in *sfl** park^Δ21^/mef2-Gal4 park^1^* animals. A replicate experiment at 23°, where Gal4 activity, and hence the degree of RNAi, was lower, reduced the lethality and also showed significant increases in ubiquitin-positive punctae in *Atg5*^*RNAi*^ bearing animals, although the punctae were fewer and less bright (*t-test*, *P* = 0.01). The elevated levels of punctae required both *parkin* alleles to be present. These findings demonstrate that an intact autophagy system is required for the full rescue of *parkin* mutants mediated by reductions in *sfl* function.

## Discussion

### Heparan sulfate biosynthesis levels and the regulation of autophagy

Earlier work established that compromising heparan sulfate biosynthesis in *Drosophila* produces elevated levels of autophagy in larval muscle and fat body, the latter being a critical energy storage and metabolic sensing organ(Reynolds-Peterson *et al.* 2017). In studies presented here, we examined if heparan sulfate modified proteins have a broader, organism-wide role in regulating autophagy, and affect physiological processes known to be autophagy-dependent. We also examined heparan sulfate-dependent autophagy in the adult brain using two assays, autophagy-dependent cleavage of Atg8a, a critical autophagy component, as well as the levels of insoluble ubiquitin-modified proteins (IUPs) in the adult brain after ROS exposure. IUPs show age-dependent accumulation and levels of these proteins are affected by both autophagy function and exposure to ROS(Simonsen *et al.* 2008). In both of these experimental systems, reducing heparan sulfate biosynthesis showed the hallmarks of increased autophagy, elevated cleavage of Atg8a and reduced levels of IUP with ROS treatment. These findings demonstrate the capacity of heparan sulfate levels to affect autophagy in the brain and together with earlier work indicate that heparan sulfate modified protein-mediated regulation of autophagy is widely represented in many different tissues and developmental stages.

Previous studies demonstrated that the lethality associated with severe or complete loss of *sfl* or *ttv* function could be partially suppressed by reducing autophagy pathway function (Reynolds-Peterson *et al.* 2017). Those findings supported the conclusion that one of the detriments to survival produced by loss of heparan sulfate biosynthesis was mediated by abnormally elevated autophagy. Here we show that modest reductions in heparan sulfate biosynthesis are indeed not lethal, but actually increase lifespan and resistance to ROS stress. The important distinction between these two experiments is the degree of change in heparan sulfate structure and function. We posit that changing heparan sulfate structure to a modest degree that does not compromise some of the vital functions of heparan sulfate modified proteins and allows the beneficial effects of removing inhibitory activities on cellular processes, such as increased autophagy, to become apparent. In short, these pathways, both heparan sulfate biosynthesis and autophagy, are not all or none switches but can have very different effects depending on their level of function.

### Heparan sulfate biosynthesis effects on lifespan and resistance to oxidative stress

Autophagy has profound consequences on the physiology of the organism as a whole. Increased autophagy is associated with extended lifespan in a number of model organisms, including yeast, *C. elegans*, *Drosophila* and mice (Kapahi *et al.* 2017; Simonsen *et al.* 2008; Hansen *et al.* 2018; Fernández *et al.* 2018). Expression of autophagy genes and proteins show age-dependent reduction in virtually all model organisms examined. In humans, mutations affecting autophagy components are associated with a number of disorders affecting cell health and viability, suggesting that the functional connection between autophagy, ageing and age-dependent diseases is conserved (Hansen *et al.* 2018; Yen and Klionsky 2008; Fernández *et al.* 2018). Given the effect of elevated autophagy on ageing we examined the lifespan of fruit flies heterozygous for genes encoding critical enzymes of heparan sulfate biosynthesis and sulfation. Reducing the function of *ttv* or *sfl* all increased lifespan significantly, providing evidence that compromising processes supported by heparan sulfate modified proteins was protective to a physiologically relevant degree and consistent with broadly elevated levels of autophagy.

ROS can contribute to cell damage and molecular systems designed to remove these molecules provide important cell protective mechanisms. Autophagy has a number of roles in mitigating oxidative stress. First, a principal source of ROS, mitochondria, is removed by an autophagy-dependent mechanism, mitophagy. In particular, damaged mitochondria, that produce higher levels of ROS, are tagged for removal and lysosomal degradation mediated by autophagocytosis. Autophagy is also able to remove damaged proteins and protein aggregates that can result from ROS damage. It therefore follows that increases in autophagy can provide a level of resistance to ROS exposure, and this has been demonstrated experimentally. In *Drosophila* adding hydrogen peroxide to food media is lethal over a several day course and can be used to assess sensitivity to oxidative stress. Reductions in heparan sulfate biosynthesis mediated by heterozygosity for mutations in any of three genes significantly increased survival upon exposure to hydrogen peroxide, consistent with protection afforded by increases in autophagy. This is strong evidence that modulation of heparan sulfate synthesis has a broad impact on autophagy and physiology governed by this important cellular process.

Resistance to ROS stress was achieved in animals heterozygous for mutations in key heparan sulfate biosynthetic enzyme-encoding genes. Complete loss of function of these genes is lethal, affecting signaling mediated by many essential patterning molecules, including Wingless, Hedgehog and FGF. Heterozygosity for mutations in these genes does not however produce any overt morphological deficits, and they are classified as recessive mutations. Our work demonstrates that a 50% reduction in gene function however, does have physiological consequences. To understand the impact of reducing the function of heparan sulfate biosynthesis critical genes in the intact animal we have examined the structure and levels of the heparan sulfate chains in animals heterozygous for *sfl* or *ttv*. We detected changes in both amount (*ttv* and *sfl**)* and sulfation state (*sfl*). In *ttv* heterozygotes the level of heparan sulfate is reduced by 35–40% in adult animals, and *sfl* heterozygosity reduced total heparan sulfate by around 20% and also lowers the degree of sulfation. These measurable, but modest, changes in heparan sulfate levels and sulfation can clearly have a profound effect on the biology of the animal, suggesting that pharmaceutical intervention that could significantly increase autophagy does not require abrogation of heparan sulfate synthesis or modification. Similar reductions in heparan sulfate levels are observed in mice heterozygous for *Ext1* or *Ext2*, two homologs of *ttv*, indicating that the effect of partial reductions in heparan sulfate biosynthetic function on heparan sulfate levels is conserved across diverse species (Zak *et al.* 2011).

### Suppression of retinal abnormalities produced by ectopic expression of Presenilin

To evaluate the capacity of altered heparan sulfate modifications to provide cellular protection in a model of a neurodegeneration associated with AD, we overexpressed Presenilin in the nervous system of adult fruit flies. This model has been shown previously to disrupt the patterning of the retina and produce cell loss of photoreceptor neurons (Greeve *et al.* 2004; Ye and Fortini 1999). Reducing heparann sulfate biosynthesis by RNAi of either *sfl* or *ttv* rescued the reduction in retinal size, a measure of presenilin-mediated cell loss, and RNAi of *sfl* was also able to rescue the patterning abnormalities. These findings demonstrate that reducing heparan sulfate production is sufficient to protect cells from a neurotoxic and neuropatterning insult.

### Reductions in heparan sulfate biosynthesis suppress cell degeneration mediated by parkin in flight muscle

One arm of autophagy is the recognition, engulfment and lysosomal destruction of damaged mitochondria, a process known as mitophagy. Activation of autophagy can, therefore, increase the mitophagic capacity of cells. Previous work has shown that reductions of heparan sulfate biosynthesis or sulfation in muscle cells produces a reduction in mitochondrial density. Autophagosome membranes surrounding mitochondria were readily visualized in animals with compromised heparan sulfate biosynthesis, supporting the model that increased mitophagy is taking place (Reynolds-Peterson *et al.* 2017). These findings suggested that heparan sulfate-mediated regulation of autophagy can affect mitophagy. Therefore, we sought to determine if reducing heparan sulfate-biosynthesis can affect cellular loss mediated by defects in mitochondrial surveillance and removal. *parkin* is the *Drosophila* homolog of PARK2, a human gene responsible for a form of juvenile onset Parkinson’s disease (Pesah *et al.* 2004). Park protein is a ubiquitin ligase responsible for the tagging of damaged mitochondria for autophagosome-lysosomal destruction. Loss of *park* results in age-dependent degeneration of *Drosophila* flight muscles, with associated accumulation of dysmorphic mitochondria (Cackovic *et al.* 2018; Cornelissen *et al.* 2018; Vincow *et al.* 2013). Reducing the gene dosage of *sfl* had a remarkable capacity to rescue muscle cell death mediated by *park* mutations, affecting actin morphology, accumulation of ubiquitin, and dysmorphology of mitochondria as well as the key measure of flight muscle function, the capacity to fly. Reductions of *ttv* function also rescued muscle abnormalities of *park* mutants. These *in vivo* interactions demonstrate the capacity of heparan sulfate levels and structure to rescue cell degeneration in a model of a human neurodegenerative disorder.

The ability of reductions in *sfl* function to rescue muscle abnormalities in *parkin* mutants was dependent on an intact autophagy system (Figure 9). RNAi of a key autophagy gene, *Atg5*, increased ubiquitin-positive accumulations in muscle, a characteristic of *parkin* mutants. These findings support the model that changes in heparan sulfate structure affect cell survival via alterations in autophagy levels. However, changes in heparan sulfate may be affecting other biological responses that have an impact on cell physiology in *parkin* mutants; our data indicate autophagy is an important part of that response.

### Mechanism of heparan sulfate-mediated regulation of autophagy

Regulation of autophagy is complex and includes transcriptional, post-transcriptional and post-translational mechanisms (Feng *et al.* 2015). Target of Rapamycin (TOR) is a master regulator of autophagy, affecting both the activity of key Atg gene transcription factors as well as the activity of autophagosome assembly proteins. Tor activity serves to suppress autophagy and growth conditions that favor Tor activation result in reduced levels of autophagic flux. Heparan sulfate modified proteins play critical roles in signaling pathways that lead to Tor activation via PI3 kinase and this is one potential mechanism of heparan sulfate-mediated regulation of autophagy. Autophagy can also be activated by accumulation of unfolded proteins and the subsequent unfolded response (UPR) cascade. Earlier we examined one element of the UPR, the IRE-mediated splicing of Xbp (Ryoo *et al.* 2007) and found that reductions of heparan sulfate biosynthesis had no effect on this measure of the UPR. In addition, third instar larval muscles from animals bearing *ttv* mutations show lowered levels of BiP (Ren *et al.* 2009), a protein that is typically elevated upon unfolded stress responses (Pobre *et al.* 2018). Interestingly, *sfl* or *ttv* mutant larvae also show increased levels of stimulus-dependent endocytosis in the motoneuron, emphasizing that the effects of heparan sulfate structure on membrane trafficking are not limited to autophagy (Ren *et al.* 2009).

This work documents the broad inhibitory effect of heparan sulfate modification on autophagy in *Drosophila*. Studies in mice suggest this regulatory relationship is represented in vertebrates as well. Heparanase (Hpa) is an endo-D-glucuronidase that cleaves heparan sulfate and transgenic mice with ectopic expression of Hpa exhibit increased autophagy levels in multiple tissues. Conversely, knockouts of Hpa results in autophagic suppression (Ilan *et al.* 2015; Shteingauz *et al.* 2015). Collectively, these findings suggest heparan sulfate modified protein suppression of autophagy is evolutionarily conserved and occurs in many cell types and tissues in mice.

### Implications of heparan sulfate-mediated regulation of autophagy in human neurodegenerative disorders

The capacity of autophagy to remove protein aggregates and damaged mitochondria provides a means of protecting cells against pathological events that lead to cell death (Gil and Rego 2008; Komatsu *et al.* 2006a; Ramesh and Pandey 2017; Wager and Russell 2013). Activation of autophagy has the potential to be protective for neurodegeneration and conversely, suppression of autophagy confers susceptibility to cell loss. Interestingly, lysosomal storage disorders, where deficits in certain degradative enzymes result in accumulation of heparan sulfate, produce suppression of autophagy and neuronal loss (Bartolomeo *et al.* 2017; Fiorenza *et al.* 2018; Settembre *et al.* 2008a; Settembre *et al.* 2008b). A number of human neurodegenerative disorders, including AD, Huntingtin’s, and Amyotrophic Lateral Sclerosis, show protein aggregation and accumulation. Deficits in mitochondrial turnover and clearance are also implicated in neurodegeneration, Parkinson’s Disease being a well-studied case in point (Buhlman 2017; Nakamura *et al.* 2013; Trempe and Fon 2013). The data presented here demonstrates that modulation of heparan sulfate synthesis has the capacity to rescue cell loss in two models of human neurodegenerative diseases, one mediated by overexpression of Presenilin and the other by loss of *parkin* function. Overexpression of Presenilin phenocopies loss-of-function mutants in *Drosophila* suggesting this model works via a dominant-negative mechanism. Given that the majority of human AD pathogenic mutations affecting *PSEN1* reduce or eliminate γ-secretase function, the fly model may provide a reasonable assay for Presenilin-mediated pathology. Other experiments described here also show that reducing heparan sulfate biosynthetic capacity can rescue cell pathology produced by reductions of *parkin* function. Parkin, the fly homolog of PARK2, participates in mitochondrial surveillance via the ubiquitin-modification of outer membrane proteins, tagging them for mitophagy and degradation in the lysosome. Our experiments show that reducing heparan sulfate can suppress the muscle abnormalities in *parkin* mutants, including the restoration of mitochondrial morphology, and suggests that deficits in mitochondrial surveillance can be rescued by, increasing autophagy. Heparan sulfate biosynthesis may well be a useful target for intervention to increase autophagy and mitophagy, providing some protection in a variety of neurodegenerative disorders.

Earlier work has implicated heparan sulfate modified proteins in the deposition or clearance of amyloid deposits in the brain. Two studies support the proposal that reducing heparan sulfate decreases amyloid deposition in mouse models of AD. Conditional knockout of *Ext1* in post-natal neurons dramatically reduced the levels of amyloid plaques in an APP/PS1 mouse model (Liu *et al.* 2016). Similarly, overexpression of heparanase, a heparan sulfate degradative enzyme, also reduced amyloid burden in a mouse AD model (Jendresen *et al.* 2015). Heparan sulfate modified proteins are found in amyloid plaques and may play a significant role in both amyloid β deposition or clearance. Our findings suggest reduction in heparan sulfate modification could also affect autophagy, which does show alterations in AD. Autophagy is impaired in AD (Nixon and Yang 2011) and mechanisms that restore it to normal levels could be protective. Certainly, the capacity of heparan sulfate modified proteins to suppress autophagy has implications for understanding how reductions in the levels of heparan sulfate modification affect processes leading to neurodegeneration.
